# Maximum common property: a new approach for molecular similarity

**DOI:** 10.1186/s13321-020-00462-3

**Published:** 2020-10-09

**Authors:** Aurelio Antelo-Collado, Ramón Carrasco-Velar, Nicolás García-Pedrajas, Gonzalo Cerruela-García

**Affiliations:** 1 University of Informatics Science, Carretera San Antonio de los Baños Km. 2 1/2 , Boyeros, La Habana, Cuba, Havana, Cuba; 2 Department of Computing and Numerical Analysis, University of Cordoba, Campus de Rabanales, Albert Einstein Building, E-14071 Córdoba, Spain

**Keywords:** Maximum common property, Electrotopographic state index, Molecular similarity, Tanimoto function, Maximum common structure

## Abstract

The maximum common property similarity (MCPhd) method is presented using descriptors as a new approach to determine the similarity between two chemical compounds or molecular graphs. This method uses the concept of maximum common property arising from the concept of maximum common substructure and is based on the electrotopographic state index for atoms. A new algorithm to quantify the similarity values of chemical structures based on the presented maximum common property concept is also developed in this paper. To verify the validity of this approach, the similarity of a sample of compounds with antimalarial activity is calculated and compared with the results obtained by four different similarity methods: the small molecule subgraph detector (SMSD), molecular fingerprint based (OBabel_FP2), ISIDA descriptors and shape-feature similarity (SHAFTS). The results obtained by the MCPhd method differ significantly from those obtained by the compared methods, improving the quantification of the similarity. A major advantage of the proposed method is that it helps to understand the analogy or proximity between physicochemical properties of the molecular fragments or subgraphs compared with the biological response or biological activity. In this new approach, more than one property can be potentially used. The method can be considered a hybrid procedure because it combines descriptor and the fragment approaches. 
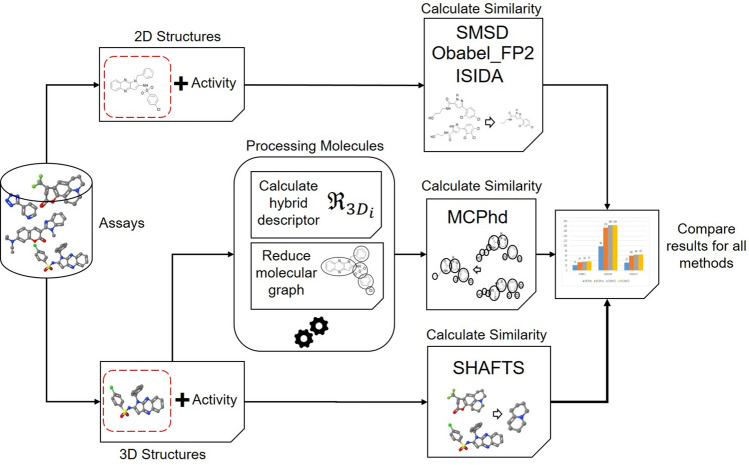

## Introduction

Molecular similarity is one of the most explored and employed concepts in cheminformatics (chemical informatics or chemoinformatics) [1]. Moreover, it is currently one of the central subjects in medicinal chemistry research [1, 2]. Molecular similarity can be evaluated using different approaches, which can be classified into two principal categories: those based on descriptors and those based on substructures [3]. To estimate similarity among molecules, it is necessary to identify those structural or chemical/physical properties that are useful to correlate and then predict the relationships among them.

Similarity calculations based on molecular descriptors use fingerprint representations [3, 4]. These representations can be codified both by topological or topographic descriptors. Topological descriptors are the most popular because the 2D representation of molecules is computationally less difficult to work with than the 3D representation [1].

This work proposes a different approach in contrast with what is rigorously known as molecular similarity or chemical similarity [1]. The descriptor and the method of reduction of the graph used contain both structural and chemical-physical information. Thus, the approach allows evaluations and comparisons to be made by accounting for not only the structure but also other properties associated with the electrostatic nature of the molecule or fragment. The methods of structural similarity in 2D are more popular and simple. However, when working with only the topology of the molecules, most of the information associated with the spatial distribution is lost, except in the molecules that are essentially flat. As opposed to 2D methods, 3D methods consider that the properties of molecules tend to be strongly associated with the spatial distribution of their atoms [5, 6]. On the other hand, the 3D methods based on 3D data usually compute a single conformation per molecule, which may not agree with the bioactive conformation. It is a common problem for all methods based on single conformation.

This issue causes a dilemma for researchers: losing all three-dimensional information for the sake of simplicity in the calculations or complicating the calculations and possibly delaying the results. The possibility of obtaining large data sets is an unquestionable reality. In that case, the eventual distortion of the 3D results due to not adjusting to the required conformation must be compensated by the increase in the number of compounds. However, such voluminous processing is not currently an impediment in terms of computational cost [7, 8].

Another concept that has been used for more than two decades is the scaffold and, more recently, scaffold hopping. These concepts allow the reduction of the molecule by eliminating R-substituents from the nucleus supposedly responsible for the activity in series of compounds in the first case, and in the other case, they allow the scaffold to be determined and enable comparisons to be made between structurally different compounds [9]. In other words, this approach bears a certain similarity to the proposed method since both seek to identify structurally different compounds that may show similar biological activity.

For these reasons, the proposed similarity method is based on the molecular description with a 3D descriptor that has structural information and on the polarity of the molecular graph or its fragments defined by a chemical graph reduction method.

Furthermore, molecular similarity based on substructure allows obtaining the molecular fragment or common subgraph among pairs of compounds [10, 11]. Several similarity methods have been developed based on a group of algorithms aimed at obtaining the largest common subgraph among a pair of compounds, the maximum common subgraph (MCS) [12–14]. To quantify the molecular similarity, this method uses the Tanimoto coefficient ($Tc_{MCS}$) [15, 16].

In this work, we introduce a new concept called maximum common property (MCPhd), inspired by MCS, to quantify the similarity based on substructure, using the electrotopographic state index for atoms ($Sstate_{3D}$) [17], which was developed from its parent electrotopologic defined by Kier and Hall [18] from the connectivity matrix of the hydrogen-depleted chemical graph as an atomic descriptor.

The rest of the paper is organized in sections as follows: Related Works describes several relevant and recent proposals related to this work; Materials and Methods describes the dataset and molecular codification, the general procedure and the proposed MCPhd algorithm; Results and Discussion describes the experimental results; and finally, Conclusions presents a summary of this work.

## Related works

In the SAR and QSAR approaches, the similarity between molecular structures is measured from some fragments of structural interest, physico-chemical properties, or other characteristics that are relevant to the biological activity under study. Therefore, the quality in the description and representation of molecular structures is a very important issue in the construction of computational models [19].

There are several proposals that consider the 3D information of the structure to calculate the similarity between chemical compounds. For example, Raymond and Willett [20] proposed a 3D MCSs method for similarity searching based on finding the largest set of atoms common to both molecules that preserves all pairwise distance constraints in both molecules. Although the number of freedom rotational degree is usually a difficulty, it was solved by generating several conformations. In order to establish the maximum and minimum possible distances between all pairs of atoms in a molecule, they applied the distance geometry described by Crippen et al. [21]. This procedure shows a computational complexity of $O{(N^{3}) }$.

Other 3D similarity methods like LS-align [22], generate atom-level structural alignments of ligand molecules, by an iterative heuristic search of the target function that combines inter-atom distance with mass and chemical bond comparisons.

Shape-feature similarity (SHAFTS) [23] is a hybrid approach for 3D molecular similarity calculation. The method adopts a hybrid similarity metric combined with molecular shape and colored (labeled) chemistry groups annotated by pharmacophore features for 3D similarity calculation. The method needs molecular alignments and superpositions between the target and the query molecules.

The ligand-based approach LigCSRre [24] uses 3D structural data of molecules for similarity studies. It combines a 3D maximum common substructure search algorithm independent from atom order with a tunable description of atomic compatibilities to prune the search.

3D similarity is attracting attention of the scientific community. Many methods to describe the shape of molecules have been developed. Surface-based approaches such as 3D Zernike descriptors and others demonstrated a good virtual screening performance [25]. Futhermore, nowadays there is a wide variety of web services, source code libraries and frameworks such as Open Babel [26], CoSiAn [27], ChemMapper [28], SMSD Toolkit [29], Corina [30], ISIDA-Platform [31], Chemaxon Web Services [32], and Chemical Development Kit (CDK) library [33] that allow to calculate 2D and 3D descriptors, build and validate QSAR models, and support the implementation of new computational models and algorithms.

## Materials and methods

### Sample used

We employed a set of 4-aminobicyclo[2.2.2]octan-2-yl 4-aminobutanoates (Table 1) reported by Weis et al. [34] and evaluated compounds against the multiresistant K-1 strain of Plasmodium falciparum.

**Table 1:**
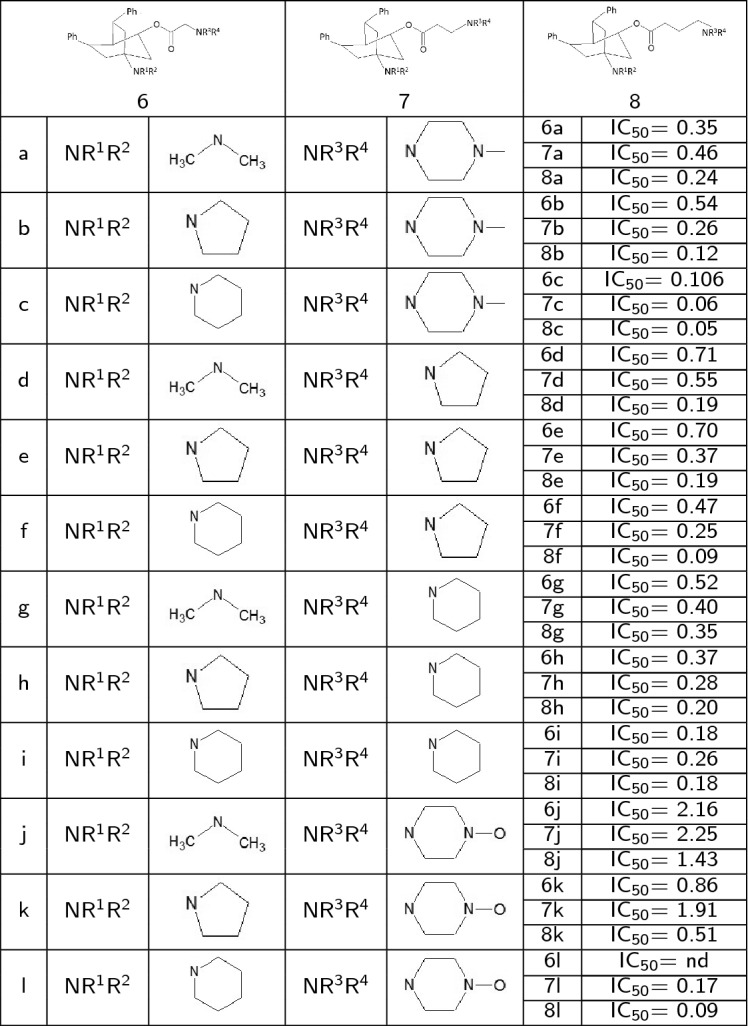
Compounds set

### Codification of structures

The electrotopographic state index for atoms [17] was used to codify chemical structures. This index is defined by Eq. (1).

1 $$\begin{aligned} Sstate_{3D}=I_{i}+\triangle I_{ij} \end{aligned}$$

where $Sstate_{3D}$ is the calculated value of the atom *i* in the corresponding molecule and $I_i$ is the intrinsic value of the atom *i* calculated with Eq. (2).

2 $$\begin{aligned} I_{i}=\left[ (2/N)2_{v}+1\right] /\delta \end{aligned}$$

where *N* is the principal quantum number of atom *I*, $\delta ^{v}$ is the number of valence electrons in the molecular skeleton ($Z^{v}$-h) and $\delta$ is the number of $\sigma$ electrons in the skeleton ($\sigma -h$). For each atom of the molecular skeleton, $\delta ^{v}$ is the number of valence electrons, $\sigma$ is the number of electrons in $\sigma$ orbitals and *h* is the number of hydrogen atoms bonded.

$\triangle I_{ij}$ represents the disturbance of the atoms of the environment, which is calculated by Eq. (3).

3 $$\begin{aligned} \triangle I_{ij}=\sum (I_{i}+I_{j})/r_{ij}^{2} \end{aligned}$$

where the sum is over the difference of the intrinsic values of atom i with respect to each one of the other atoms in the molecule and $r_{ij}^2$ is the Euclidean distance between the analyzed atoms, transforming the original topological index of Kier and Hall in topographic.

### Graph reduction

**Fig. 1:**
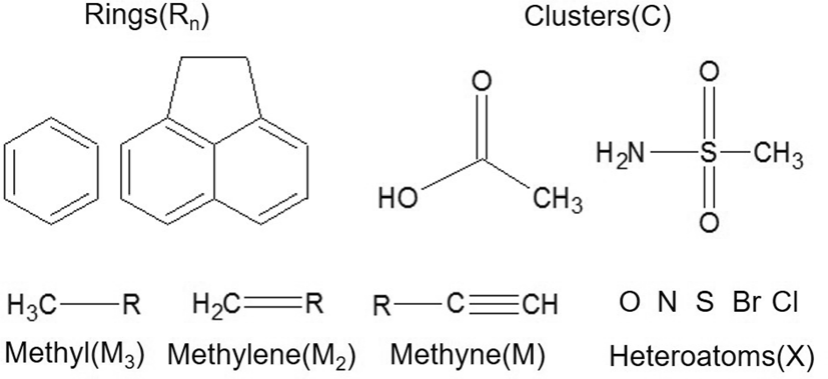
Examples of Descriptor Centers (DC) employed in the fragmentation of the chemical graphs

The reduction of the chemical graph is carried out by the method described by Carrasco et al. [35], where the descriptor centers (DCs), rings of different orders (Rn), clusters of order 3 and 4 (C3 and C4, respectively), heteroatoms such as halogens, amino, etc. (X), and terminal groups such as methyl ($M_{3}$), methylene ($M_{2}$) and methyne (M) are defined. Examples of these parameters are shown in Fig. 1. This graph reduction procedure, named CALEDE, is inspired by the procedure developed by Avindon et al. [36], where each DC is assigned the total value of $Sstate_{3D}$, quantified as the sum of the value of $Sstate_{3Di}$ of each atom that conforms to it.

### Definition of the maximum common property

The maximum common property (MCPhd) between two fully connected and complete (not hydrogen-depleted) $G_{1}$ and $G_{2}$ chemical graphs is defined as the maximum similarity in the chemical-physical properties represented by the index $Sstate_{3D}$, which exists between subgraphs $g_{1}$ and $g_{2}$ of the molecular graphs $G_{1}$ and $G_{2}$, respectively. Both $g_{1}$ and $g_{2}$ represent the link of at least two DCs that are at a Euclidean distance dE($DC_{1}$, $DC_{2}$) from their corresponding centers of mass from pairs of DCs.

To quantify the value of similarity between two compounds using the concept of the maximum common property (MCPhd), the calculation of the similarity of two compounds is assumed using the Tanimoto function or coefficient on the basis of the maximum common substructure called $Tc_{MCS}$ [15, 16]. The $Tc_{MCS}$ for two molecules A and B is defined as:

4 $$\begin{aligned} Tc_{MCS}=\frac{\left| MCS(A,B)\right| _{b}}{\left| A\right| _{b}+\left| B\right| _{b}-\left| MCS(A,B)\right| _{b}} \end{aligned}$$

where $\left| A\right| _{b}$ is the number of links of A, $\left| B\right| _{b}$ is the number of links of B and $\left| MCS(A,B)\right| _{b}$ is the number of links of the MCS of A and B. If the concept MCPhd is replaced in Eq. (4), it yields:

5 $$\begin{aligned} Tc_{MCPhd}=\frac{\left| MCPhd(A,B)\right| _{b}}{\left| A\right| _{b}+\left| B\right| _{b}-\left| MCPhd(A,B)\right| _{b}} \end{aligned}$$

where $\left| A\right| _{b}$ is the number of heavy atoms of A, $\left| B\right| _{b}$ the number of heavy atoms of B and $\left| MCPhd(A,B)\right| _{b}$ the smallest number of heavy atoms among the fragments with the highest MCPhd between A and B.

### The proposed MCPhd algorithm

**Fig. 2:**
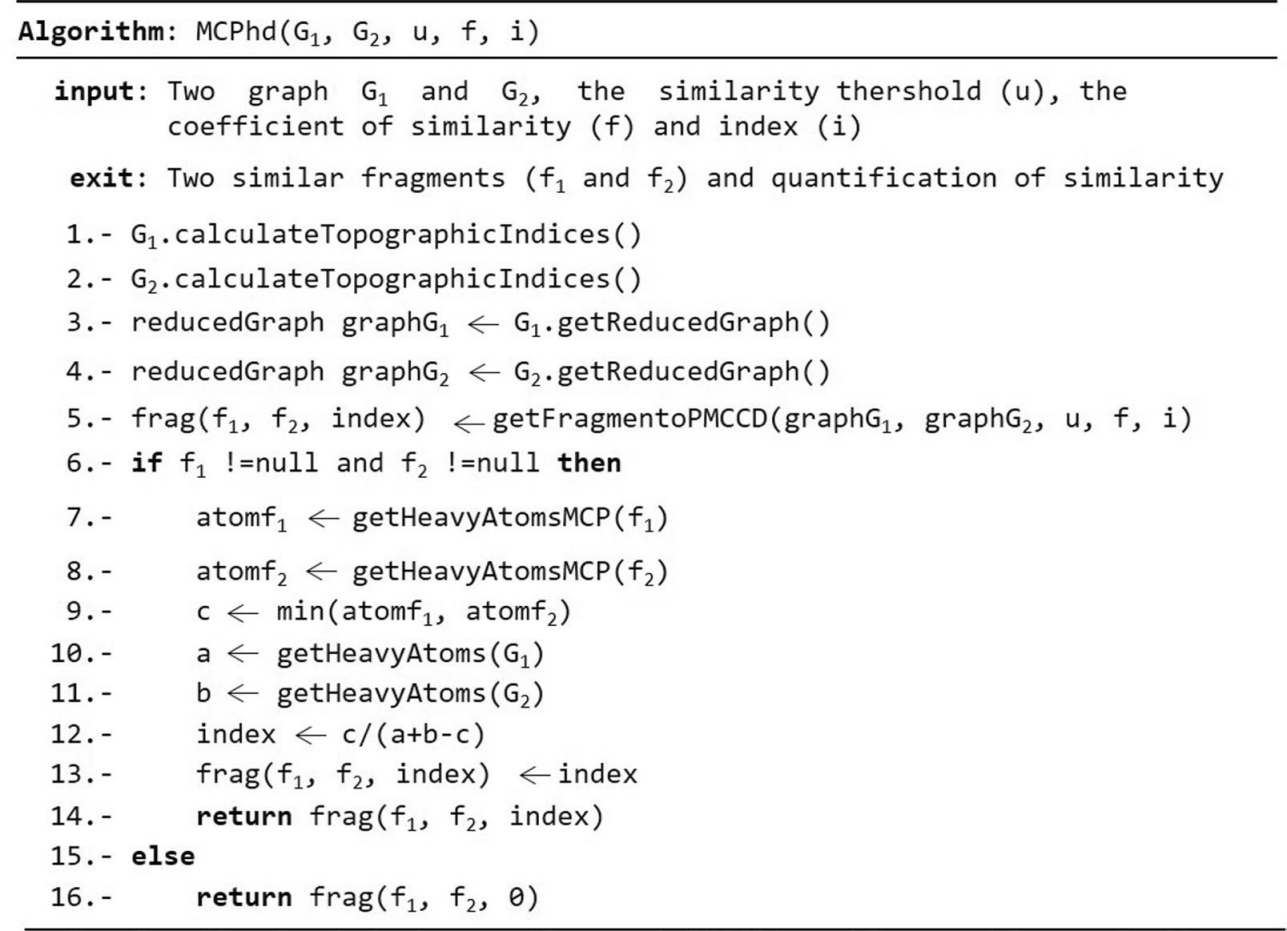
MCPhd algorithm for similarity calculation

**Fig. 3:**
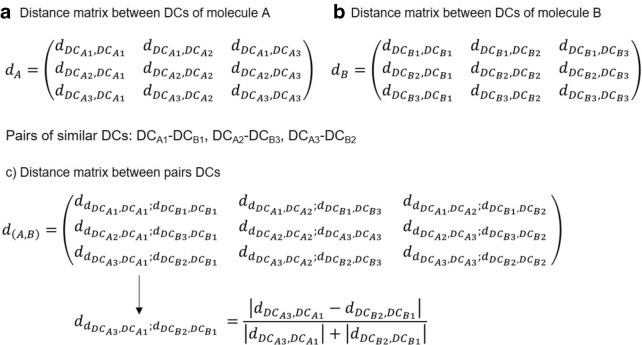
Distance matrix between pairs of similar DCs

Figure 2 shows the algorithm used for the calculation of similarity. The algorithm uses the following parameters: ($G_{1}$ and $G_{2}$) two compounds or molecules, (u) the similarity threshold, (f) the similarity coefficient and (i) the index used to quantify the similarity. First, we obtain the subgraphs ($f_{1}$ and $f_{2}$) that have a maximum common property value quantified by the index based on the parameters and similarity coefficient. These subgraphs are obtained by performing the following steps:

1. The index (i) entered as a parameter is calculated for each atom in each G_1_ and G_2_ graph using the Chemical Development Kit (CDK) library [33]. Lines 1 and 2 of the algorithm are shown in Fig. 2.
2. The graphs ($G_{1}$ and $G_{2}$) on DCs are reduced, and the total index value of each one is obtained. Lines 3 and 4 of the algorithm are shown in Fig. 2.
3. The similarity matrix between the DCs obtained from the graphs ($G_{1}$ and $G_{2}$) is constructed using the similarity coefficient introduced as a parameter, along with the distance matrix between the DCs of each graph ($G_{1}$ and $G_{2}$) using the Euclidean distance. Line 5 of the algorithm is shown in Fig. 2.
4. The DCs from each graph ($G_{1}$ and $G_{2}$) that meet the condition that the similarity value must be higher than the similarity threshold (*u*), entered as a parameter, are selected. Line 5 of the algorithm is shown in Fig. 2.
5. Finally, using the list of DCs obtained in the previous step and the distance matrices of the DCs in graph $G_{1}$ and $G_{2}$, a new distance matrix between pairs of DCs in each graph $G_{1}$ and $G_{2}$ is constructed using the Canberra distance coefficient [38], as shown in Fig. 3. Then for each pair of DCs selected, a list is created in which the pairs of DCs in the created matrix whose distance is less than or equal to 0.15 are stored. Finally, the largest lists is selected and from each one the subgraphs $f_{1}$ and $f_{2}$ are generated. Line 5 of the algorithm is shown in Fig. 2

Then, for a pair of subgraphs ($f_{1}$ and $f_{2}$) obtained and the graphs ($G_{1}$ and $G_{2}$), the values of the variables needed to quantify similarity are obtained using the similarity coefficient (u) for the discrete data entered as a parameter. Variable c is assigned the least number of heavy atoms belonging to the subgraphs ($f_{1}$ and $f_{2}$), while variables a and b are assigned the number of heavy atoms belonging to each graph ($G_{1}$ and $G_{2}$), respectively. Finally, these values are substituted in the similarity function to obtain the quantification of the similarity of the graphs (G_1_ and G_2_). Lines 6 to 16 of the algorithm are shown in Fig. 2. Furthermore, if there are several subgraphs $f_{1}$ and $f_{2}$, the same operation is performed for each one and the pair of subgraphs $f_{1}$ and $f_{2}$ with the highest similarity value is selected.

**Fig. 4:**
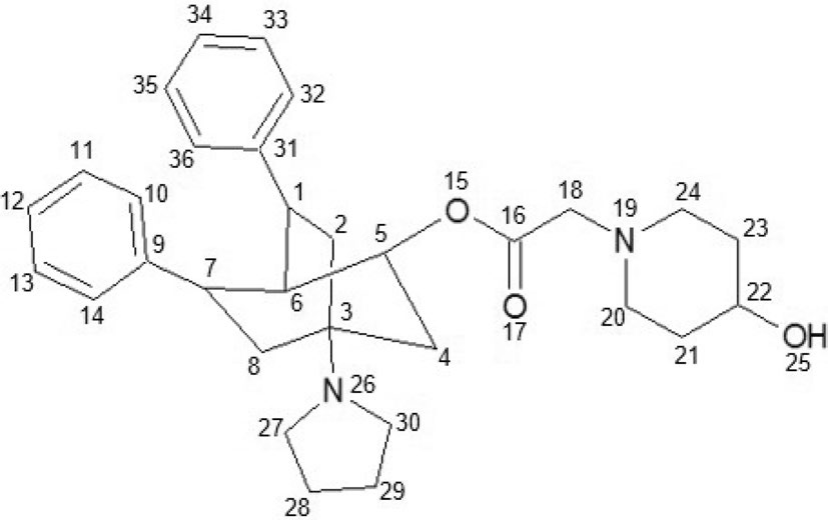
2D graph of the 6k molecule

**Fig. 5:**
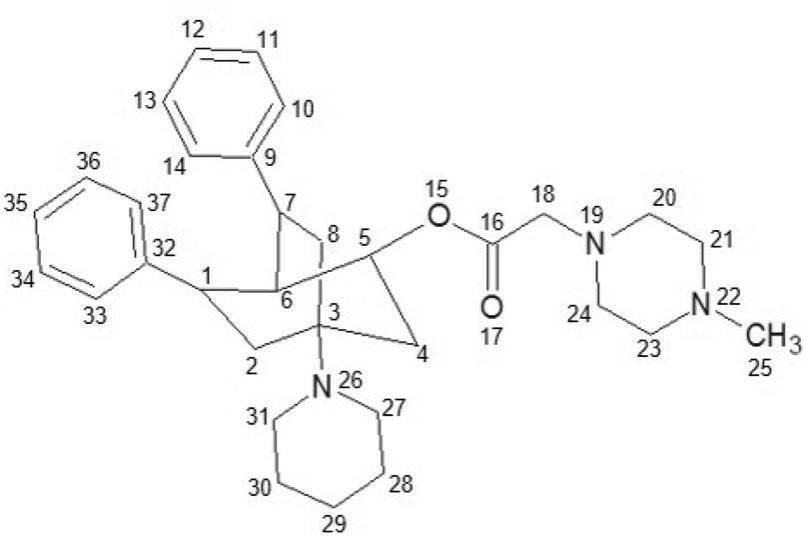
2D graph of the 6c molecule

**Fig. 6:**
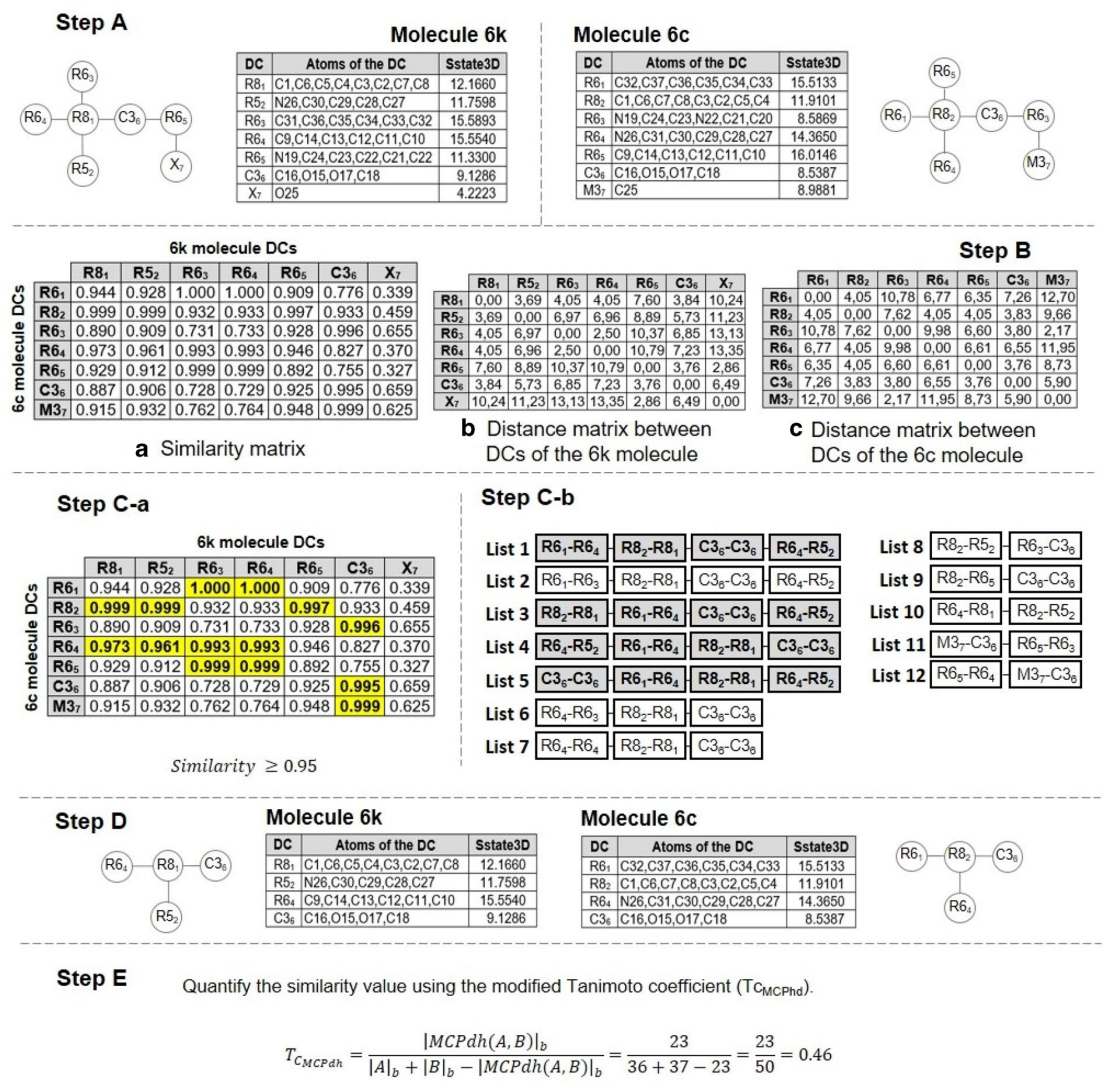
Example of applying the MCPhd algorithm to the 6k and 6c molecules belonging to the dataset

The use of the algorithm is exemplified below using the molecules 6k and 6c present in the dataset as shown in Figs. 4 , 5, respectively. We use 5 parameters $(G_{1}, G_{2}, u, f, i)$ for its operation, where $G_{1}$ and $G_{2}$ are the molecular graphs 6k and 6c respectively, (*i*) is the index ($Sstate_{3D}$), (*u*) is the similarity threshold, and (*f*) is the similarity function. For this example, we will use 0.95 and the modified Tanimoto coefficient ($Tc_{MCPhd}$) as the threshold and similarity function, respectively. Then, after assigning the parameters, the following steps are performed:

1. The $Sstate_{3D}$ index is calculated for each atom present in molecules 6k and 6c; these results are shown in Tables 2 and 3.
2. The 6k and 6c molecular graphs on DCs are reduced, and each is given the value of the total $Sstate_{3D}$ index. As shown in step A of Fig. 6, molecule 6k is reduced on the DCs ($R8_{1}$, $R5_{2}$, $R6_{3}$, $R6_{4}$, $R6_{5}$, $C3_{6}$, $X_{7}$), while molecule 6c is reduced on ($R6_{1}$, $R8_{2}$, $R6_{3}$, $R6_{4}$, $R6_{5}$, $C3_{6}$, $M_{7}$).
3. The similarity matrix between the DCs of each molecule 6k and 6c is constructed using the continuous Tanimoto coefficient (Tc) [37], together with the distance matrices between the DCs of each molecule (6k and 6c), as shown in step B of Fig. 6.
4. DCs are selected from each molecule (6k and 6c) that meet the condition that the similarity value is above the similarity threshold of 0.95. The DCs selected from molecules 6k and 6c are ($R8_{1}$, $R5_{2}$, $R6_{3}$, $R6_{4}$, $R6_{5}$ and $C3_{6}$) and ($R6_{1}$, $R8_{2}$, $R6_{3}$, $R6_{4}$, $R6_{5}$, $C3_{6}$ and $M3_{7}$), respectively, as shown in step C-a in Fig. 6. Furthermore, using the distance matrices of the graphs obtained in the previous step, for each pair of DCs, a list is constructed with the pairs of DCs that are at a Canberra distance less than or equal to 0.15, as shown in step C-b in Fig. 6.
5. From the lists of DC pairs obtained in the previous step, the following DCs are selected, namely, ($R8_{1}$, $R5_{2}$, $R6_{4}$ and $C3_{6}$) and ($R6_{1}$, $R8_{2}$, $R6_{3}$ and $C3_{4}$), corresponding to the lists (1, 3, 4 and 5) according to the larger size list with the same DCs in common.

Finally, the similarity value of the two molecules 6k and 6c is quantified using the modified Tanimoto coefficient ($Tc_{MCPhd}$), where the value of $\left| MCPhd(A,B)\right| _{b}$ is the lowest number of heavy bonds present between fragments $f_{1}$ and $f_{2}$, while the values of $\left| A\right| _{b}$ and $\left| B\right| _{b}$ are obtained from the number of heavy atoms present in molecules 6k and 6c, respectively. With these values, it is possible to quantify the similarity between molecules 6k and 6c. In step E of Fig. 6, it can be seen that the number of heavy atoms of fragments $f_{1}$ and $f_{2}$ is 23 and 24, respectively, so the value of $\left| MCPhd(A,B)\right| _{b}$ is 23, while the number of heavy atoms of molecules 6k and 6c is 36 and 37, respectively; that is, $\left| A\right| _{b}=36$ and $\left| B\right| _{b}=37$. Therefore, the calculated value of similarity between molecules 6k and 6c is 0.46.

**Table 2:** Result of the $Sstate_{3D}$ calculation for each atom of the molecule 6k

Molecule 6k					
Atom	Number	Sstate3D	Atom	Number	Sstate3D
C	1	0.27653	N	19	− 1.4592
C	2	4.24474	C	20	3.38006
C	3	− 2.11427	C	21	3.11542
C	4	4.25231	C	22	− 0.12941
C	5	0.49396	C	23	3.09432
C	6	0.52371	C	24	3.32881
C	7	0.28273	O	25	4.22234
C	8	4.2063	N	26	− 1.30847
C	9	− 0.37931	C	27	3.52697
C	10	3.51969	C	28	2.9831
C	11	2.98731	C	29	3.01061
C	12	2.89649	C	30	3.54754
C	13	2.99502	C	31	− 0.38433
C	14	3.5348	C	32	3.54001
O	15	− 0.08644	C	33	2.99825
C	16	− 0.10545	C	34	2.90069
O	17	5.5644	C	35	2.99544
C	18	3.75612	C	36	3.53921

**Table 3:** Result of the $Sstate_{3D}$ calculation for each atom of the molecule 6c

Molecule 6c					
Atom	Number	Sstate3D	Atom	Number	Sstate3D
C	1	0.35527	C	20	3.314
C	2	4.17197	C	21	2.96595
C	3	− 2.19195	N	22	− 2.22668
C	4	4.25527	C	23	2.94306
C	5	0.37013	C	24	3.25612
C	6	0.50352	C	25	8.9881
C	7	0.21375	N	26	− 1.46525
C	8	4.23211	C	27	3.52712
C	9	− 0.24053	C	28	2.94719
C	10	3.50889	C	29	2.85191
C	11	3.00512	C	30	2.95634
C	12	2.94959	C	31	3.54763
C	13	3.09722	C	32	− 0.13708
C	14	3.69435	C	33	3.3945
O	15	− 0.48635	C	34	2.9161
C	16	− 0.31876	C	35	2.85145
O	17	5.6053	C	36	2.96557
C	18	3.73854	C	37	3.52272
N	19	− 1.66555			

### Small molecule subgraph MCS approach

The Small Molecule Subgraph Detector (SMSD) algorithm differs from previous MCS algorithms in that it uses a combination of several algorithms to find the common maximum subset and filters the results in a way that is chemically relevant because it incorporates chemical knowledge (coincidence of atom type with information sensitive and insensitive to the bond) while searching for molecular similarity. In addition, the algorithm calculates the maximum subgraph common between two molecules (A and B) by combining the power of the VFLibMCS, MCSPlus and CDKMCS algorithms. These algorithms are used on a case-by-case basis, depending on the molecules under consideration for the common maximum subgraph search [29]. This algorithm is implemented in the SMSD tool available free of charge on the official site of the European Institute of Bioinformatics.

### General experimental procedure

**Fig. 7:**
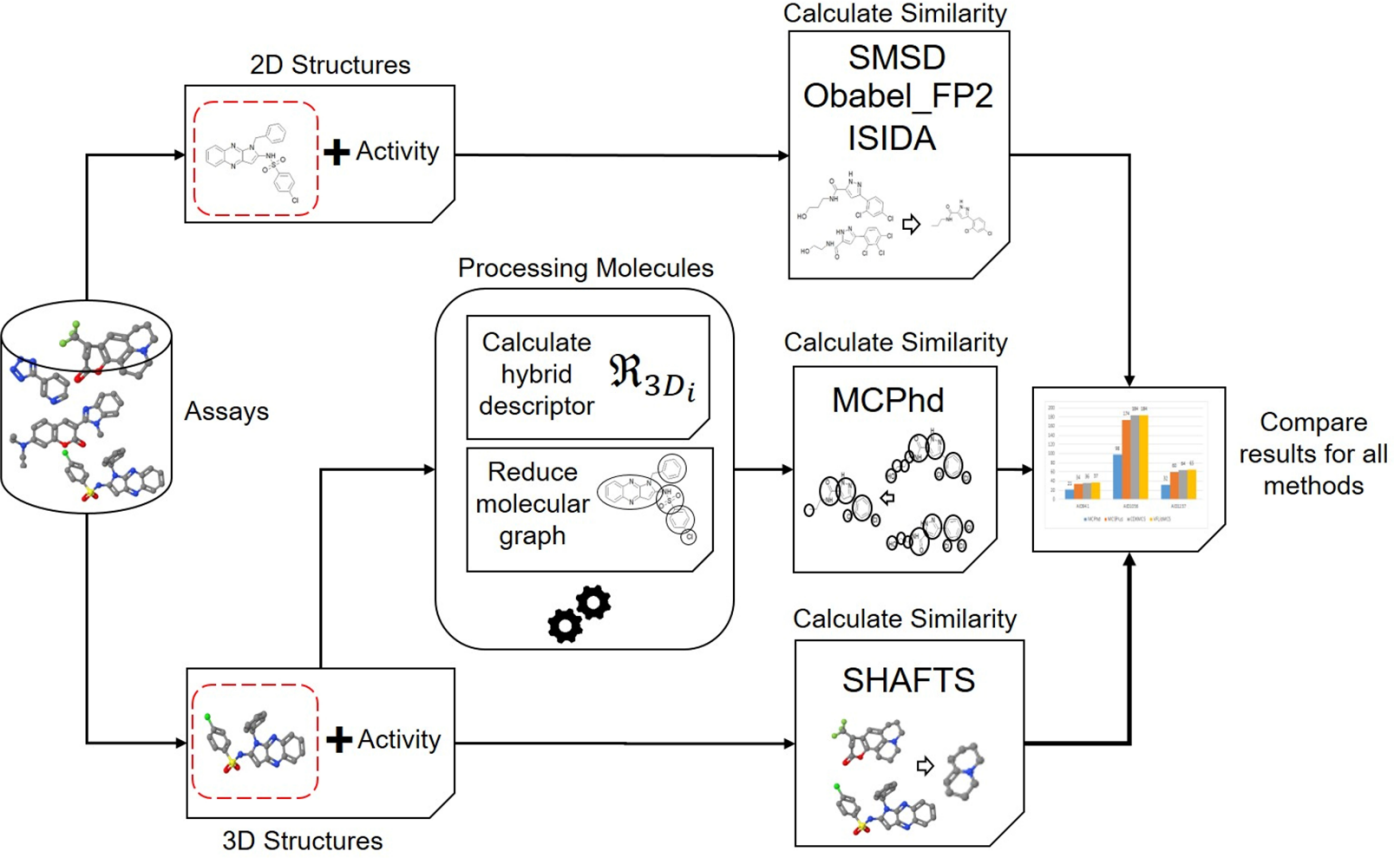
General experimental procedure

The experiments were carried out as shown in Fig. 7, based on a test of 36 compounds with a 2D structure, which have been tested experimentally in the study conducted by Weis et al. in 2014 [34]. The 3D structure of each compound was obtained through the Corina online service [30]. The 2D structures were used to calculate the molecular similarity (all against all) with the SMSD, OBabel_FP2 and ISIDA algorithms, while the 3D structures were processed to calculate the $Sstate_{3D}$ index of each atom and to reduce their graphs on DCs in order to apply the MCPhd algorithm to calculate the molecular similarity (all against all), and to use the SHAFTS method. The similarity was calculated using the OBabel_FP2, SHAFTS and ISIDA methods through the web service CoSiAn (Combinatorial Similarity Analysis) [27] and ChemMapper [28]. Additional file 1 contains all necessary data/files to reproduce the results.

To quantify the value of similarity between different $IC_{50}$ we defined the following coefficient $TcIC_{50}$ based on continuous Tanimoto [37]:

6 $$\begin{aligned} TcIC_{50}=\frac{A_{{IC}_{50}}*B_{{IC}_{50}}}{\left( A_{{IC}_{50}}\right) ^2+\left( B_{{IC}_{50}}\right) ^2-\left( A_{{IC}_{50}}*B_{{IC}_{50}}\right) } \end{aligned}$$

where $A_{{IC}_{50}}$ and $B_{{IC}_{50}}$ are the $IC_{50}$ value of A and B respectively.

Finally, the results obtained by all the algorithms were compared from different perspectives: (i) the statistical difference of the MCPhd results with respect to those obtained by the other methods; (ii) the ratio of the similarity values obtained by the different methods against the values obtained for $TcIC_{50}$; (iii) the percentage of success for the different similarity methods to find structures with the same activity, similar to a screening process; (iv) the results of the different methods in relation to the concept of bioisosterism or the analogy between the physicochemical properties of the molecular fragments; (v) the computational cost.

The MCPhd algorithm was implemented using the JAVA language and CDK library, all test were executed on an Intel(R) Core(TM) i7-7500U PC with 16 GB of RAM.

## Results and discussion

The molecular similarity methods compared in this work, SMSD OBabel_FP2, ISIDA, SHAFTS and MCPhd, use different approaches to quantify the similarity between two molecular graphs or molecules. Whereas SMSD employs graph isomorphism and no other properties associated to the molecular structure, OBabel_FP2 uses the similarity between hashed fingerprints that represent molecule substructures, ISIDA employs substructural molecular fragments, and SHAFTS adopts a hybrid similarity metric combined with molecular shape and colored chemistry groups for 3D molecular similarity calculation. The similarity calculated with MCPhd is based on the criterion of analogy or proximity between the physicochemical properties of the molecular fragments or subgraphs that are compared, expressing these properties as an $Sstate_{3D}$ value.

As we will show, this approach places MCPhd closer to the concepts of bioisosterism. Bioisosterism denote that two different molecules can afford similar biological responses if the structural features are accomplished by physicochemical property that is responsible in great measure of the biological response. This concept was coined by Friedman [39], extended by Burger [40] and recently used by Lassalas et al. [41] and Tahirova [42].

Using these different approaches, different similarity values were obtained. Table 4 shows the results of the comparison with the remaining 35 molecules of the sample, with compounds 8c and 7j used as target elements since they had the minimum and maximum $IC_{50}$ values, respectively.

**Table 4:** Molecular similarity values of the most active and inactive compounds with the rest of the dataset

Molecule 8c						Target	${{IC}}_{50}$	Molecule 7j					
OBabel_FP2	SHAFTS	ISIDA	SMSD	MSChd	${\hbox {TcIC}}_{50}$			OBabel_FP2	SHAFTS	ISIDA	SMSD	MSChd	${\hbox {TcIC}}_{50}$
1.00	1.00	1.00	1.00	1.00	1.00	8c	0.05	0.82	0.78	0.88	0.61	0.42	0.02
0.90	1.00	0.97	0.67	0.97	0.97	7c	0.06	0.92	0.82	0.90	0.83	0.43	0.03
0.91	1.00	0.97	0.95	0.77	0.74	8f	0.09	0.88	0.80	0.90	0.64	0.60	0.04
0.92	1.00	0.90	0.90	0.70	0.74	8l	0.09	0.89	0.77	0.97	0.61	0.35	0.04
0.77	0.87	0.90	0.65	0.77	0.68	6c	0.106	0.74	0.73	0.81	0.60	0.29	0.05
0.98	0.79	1.00	0.97	0.75	0.55	8b	0.12	0.80	0.88	0.88	0.62	0.33	0.06
0.82	0.84	0.88	0.67	0.71	0.37	7l	0.17	1.00	0.83	1.00	0.92	0.62	0.08
0.72	0.87	0.87	0.67	0.53	0.35	6i	0.18	0.79	0.73	0.83	0.61	0.29	0.09
0.92	1.00	0.97	0.93	0.75	0.35	8i	0.18	0.89	0.78	0.90	0.62	0.43	0.09
0.89	0.76	0.97	0.87	0.46	0.33	8d	0.19	0.86	0.91	0.90	0.68	0.64	0.09
0.90	0.93	0.97	0.92	0.56	0.33	8e	0.19	0.87	0.77	0.90	0.65	0.42	0.09
0.91	0.90	0.97	0.90	0.55	0.31	8h	0.20	0.88	0.74	0.90	0.64	0.33	0.09
0.97	0.77	1.00	0.92	0.50	0.25	8a	0.24	0.79	0.87	0.88	0.65	0.69	0.12
0.82	0.89	0.95	0.70	0.74	0.24	7f	0.25	0.99	0.77	0.92	0.87	0.51	0.12
0.88	0.82	0.97	0.65	0.69	0.23	7b	0.26	0.90	0.76	0.90	0.85	0.33	0.13
0.82	1.00	0.95	0.69	0.73	0.23	7i	0.26	1.00	0.82	0.92	0.89	0.44	0.13
0.82	1.00	0.95	0.67	0.53	0.21	7h	0.28	0.99	0.83	0.92	0.92	0.34	0.14
0.73	0.71	0.90	0.59	0.46	0.16	6a	0.35	0.69	0.87	0.81	0.64	0.64	0.18
0.91	0.74	0.97	0.85	0.45	0.16	8g	0.35	0.88	0.88	0.90	0.67	0.71	0.18
0.71	0.82	0.87	0.61	0.37	0.15	6h	0.37	0.78	0.75	0.83	0.63	0.43	0.19
0.80	0.73	0.95	0.68	0.54	0.15	7e	0.37	0.97	0.94	0.92	0.89	0.35	0.19
0.82	0.74	0.95	0.62	0.43	0.14	7g	0.40	1.00	0.84	0.92	0.97	0.73	0.21
0.87	0.71	0.97	0.61	0.61	0.12	7a	0.46	0.88	0.91	0.90	0.89	0.71	0.24
0.71	0.90	0.87	0.68	0.54	0.12	6f	0.47	0.78	0.75	0.83	0.63	0.46	0.25
0.91	0.91	0.90	0.88	0.54	0.11	8k	0.51	0.88	0.72	0.97	0.62	0.35	0.27
0.70	0.71	0.87	0.60	0.38	0.11	6g	0.52	0.77	0.88	0.83	0.66	0.66	0.28
0.76	0.76	0.90	0.63	0.56	0.10	6b	0.54	0.73	0.83	0.81	0.61	0.31	0.29
0.80	0.75	0.95	0.64	0.44	0.10	7d	0.55	0.97	0.87	0.92	0.94	0.74	0.30
0.70	0.86	0.87	0.62	0.38	0.08	6e	0.70	0.77	0.77	0.83	0.64	0.33	0.40
0.68	0.74	0.87	0.61	0.39	0.08	6d	0.71	0.75	0.91	0.83	0.68	0.68	0.40
0.71	0.70	0.81	0.63	0.36	0.06	6k	0.86	0.78	0.85	0.90	0.61	0.37	0.50
0.92	0.75	0.90	0.83	0.42	0.04	8j	1.43	0.89	0.85	0.97	0.65	0.65	0.83
0.82	0.70	0.88	0.65	0.52	0.03	7k	1.91	0.99	0.99	1.00	0.95	0.50	0.97
0.71	0.70	0.81	0.59	0.38	0.02	6j	2.16	0.78	0.86	0.90	0.64	0.64	1.00
0.82	0.78	0.88	0.61	0.42	0.02	7j	2.25	1.00	1.00	1.00	1.00	1.00	1.00

To determine whether the results produced by the MCPhd methods are significantly different, a non-parametric statistical test is used for two independent Mann-Whitney samples [43] with a significance level of 5%. The results of the Mann-Whitney U statistic and *p* values (bilateral asymptotic significance) for the most active (8c) and least active (7j) compounds are shown in Table 5. As the *p* values for both compounds are below 0.05, it is concluded that the similarity values obtained by the MCPhd methods were significantly different from the other similarity methods.

**Table 5:** Mann-Whitney test values between the MCPhd method with the remainder on the most active and inactive compounds in the dataset

Methods	Molecule 8c		Molecule 7j	
	U of Mann-Whitney	p (Sig. Asint. Bilateral)	U of Mann-Whitney	p (Sig. Asint. Bilateral)
MCPhd				
OBabel_FP	131.50	0.00	33.00	0.00
SHAFTS	117.00	0.00	34.50	0.00
ISIDA	70.50	0.00	33.50	0.00
SMSD	245.50	0.00	98.00	0.00

**Fig. 8:**
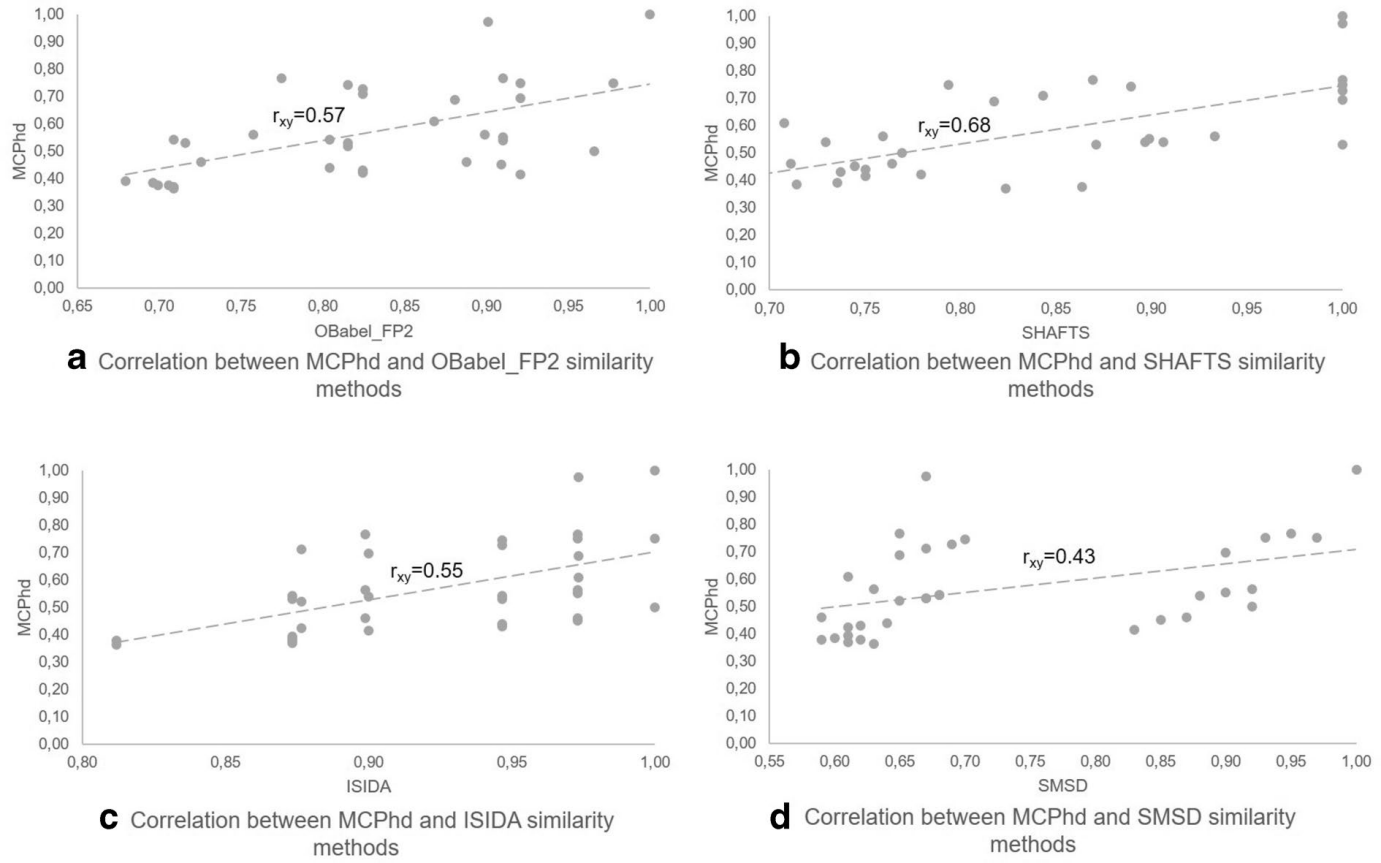
Correlation between the similarity of MCPhd and the rest of methods using the compound 8c as reference

**Fig. 9:**
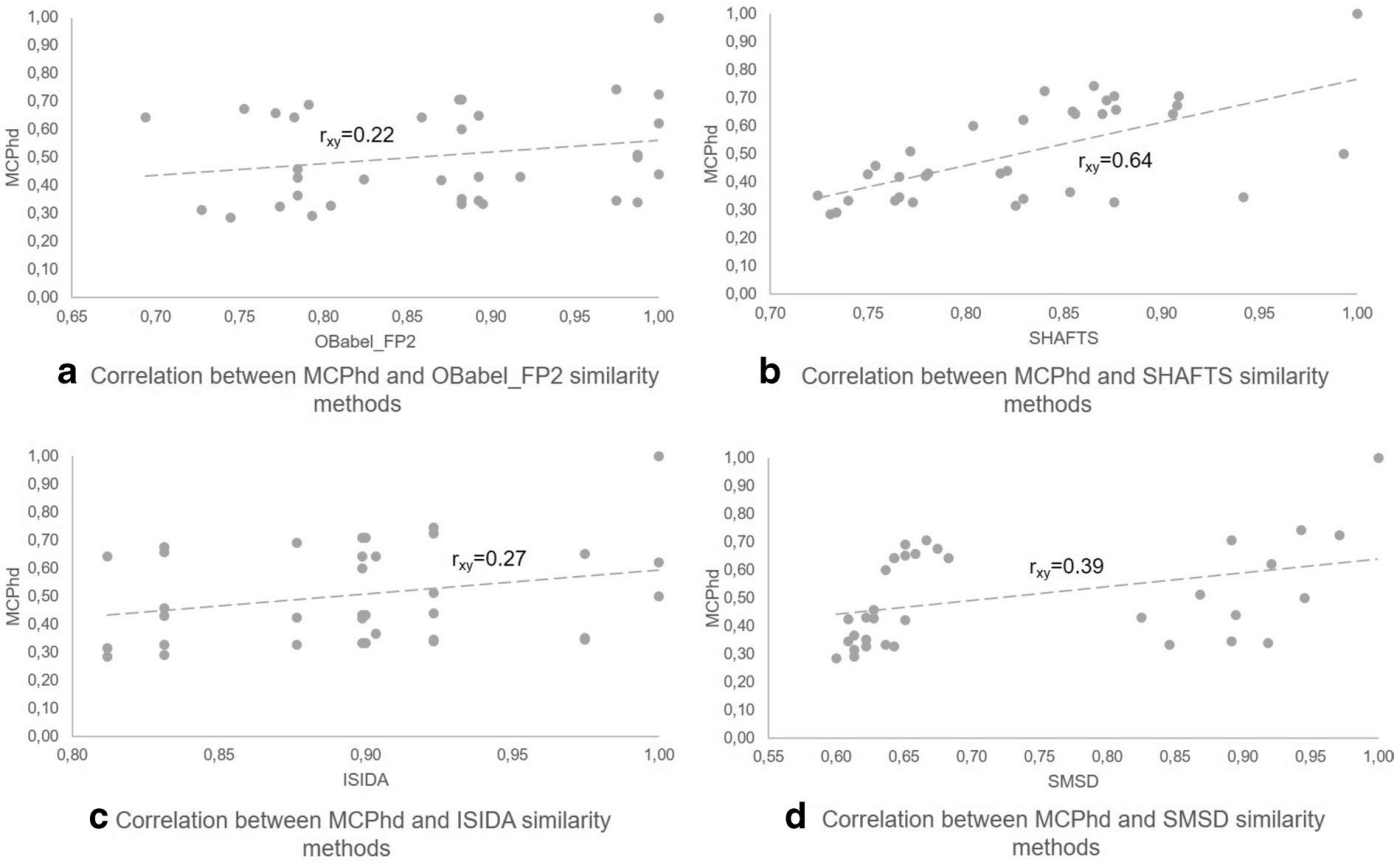
Correlation between the similarity of MCPhd and the rest of methods using the compound 7j as reference

Furthermore, it can be seen in Figs. 8, 9 for the most active compound (8c) and the least active compound (7j), respectively, that the results obtained by the similarity methods used showed a low correlation with the results achieved when applying the MCPhd method. Table 6 shows these results for all the active compounds in the dataset.

**Table 6:** Correlation results between MCPhd and the rest of methods for the active compounds in the dataset

Molecule	${\hbox {IC}}_{50}$	Correlation			
		${\hbox {r}}_{\mathrm{xy}}$ a	${\hbox {r}}_{\mathrm{xy}}$ b	${\hbox {r}}_{\mathrm{xy}}$ c	${\hbox {r}}_{\mathrm{xy}}$ d
8c	0.05	0.57	0.68	0.55	0.43
7c	0.06	0.50	0.69	0.47	0.43
8f	0.09	0.44	0.62	0.24	0.40
8l	0.09	0.45	0.71	0.33	0.53
6c	0.106	0.45	0.50	0.40	0.44
8b	0.12	0.49	0.05	0.36	0.35
7l	0.17	0.44	0.42	0.38	0.47
6i	0.18	0.39	0.46	0.37	0.45
8i	0.18	0.48	0.82	0.50	0.48
8d	0.19	0.26	0.00	0.20	0.30
8e	0.19	0.31	0.42	0.14	0.28
8h	0.20	0.38	0.41	0.30	0.40
8a	0.24	0.42	0.19	0.26	0.40
7f	0.25	0.34	0.33	0.15	0.41
7b	0.26	0.50	− 0.02	0.42	0.37
7i	0.26	0.28	0.76	0.22	0.46
7h	0.28	0.26	0.49	0.14	0.34

a-OBabel_FP2 vs MCPhd, b-SHAFTS vs MCPhd,

c-ISIDA vs MCPhd and d-SMSD vs MCPhd

As the maximum inhibitory concentration ($IC_{50}$) is a measure of a compound’s efficacy in inhibiting biological or biochemical function, it is expected that compounds with near values of $IC_{50}$, are very similar and with far values of $IC_{50}$, the compounds will exhibit very low similarity. Under that hypothesis, the results were analyzed from another perspective. The similarity was calculated for the values of the variable $IC_{50}$ of the most active compound (8c) and the less active compound (7j) against the rest of the dataset. The results are shown in the $TcIC_{50}$ columns of Table 4.

**Fig. 10:**
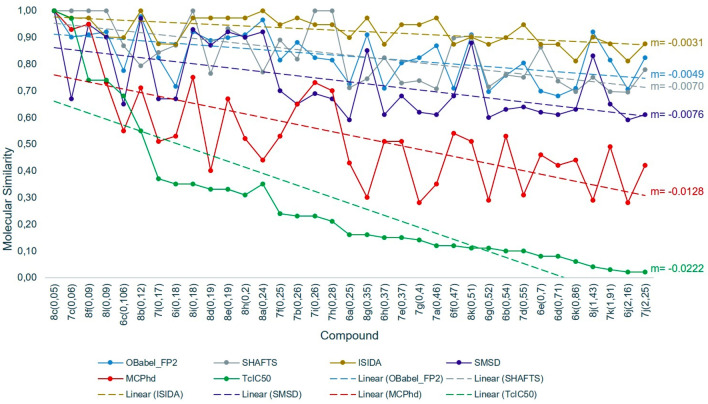
Comparison of the molecular similarity between compound 8c and the rest of the dataset

**Fig. 11:**
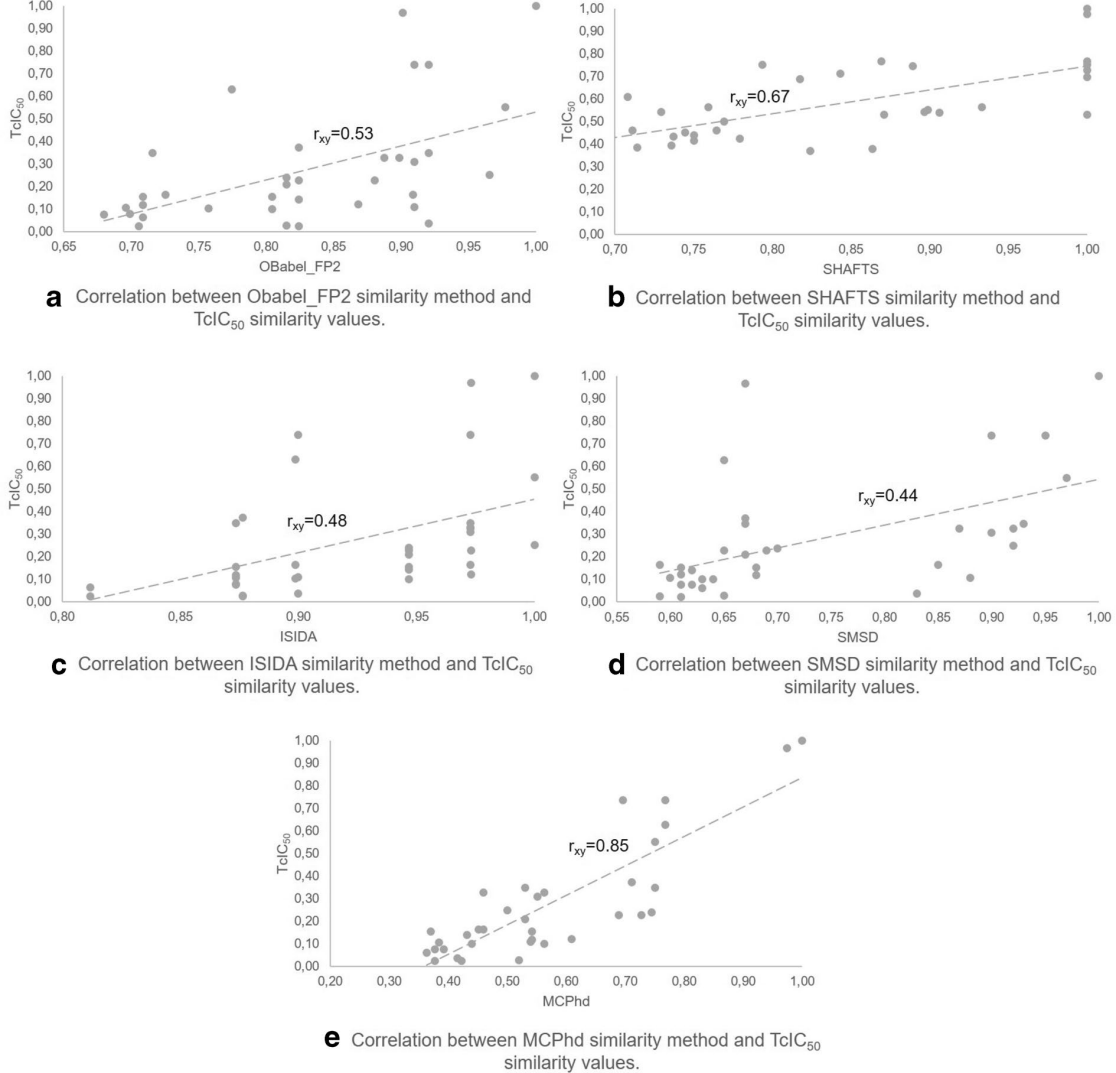
Correlation between similarity methods and $TcIC_{50}$ similarity values between compound 8c and the rest of the dataset

Subsequently, the molecular similarities calculated by the different methods were compared with this new variable. As shown in Fig. 10, the similarity results obtained by the MCPhd method for the most active compound (8c) had a slope closer to that obtained with the $TcIC_{50}$ similarity; furthermore, the results were better correlated obtained the MCPhd method the best Pearson correlation coefficient ($r_{xy}$ = 0.85) compared to the remaining methods as Fig. 11 shows.

**Fig. 12:**
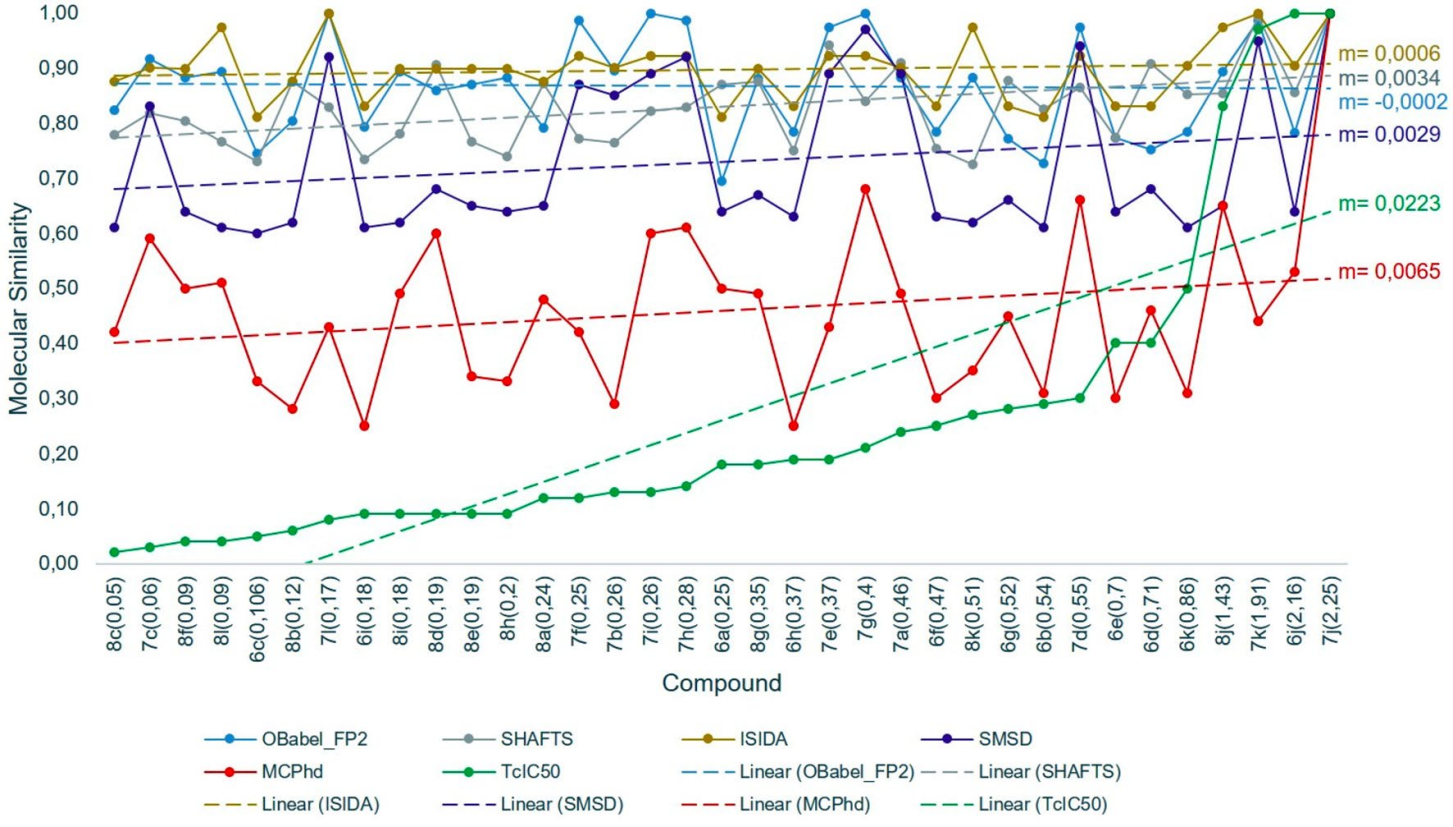
Comparison of the molecular similarity between compound 7j and the rest of the dataset

**Fig. 13:**
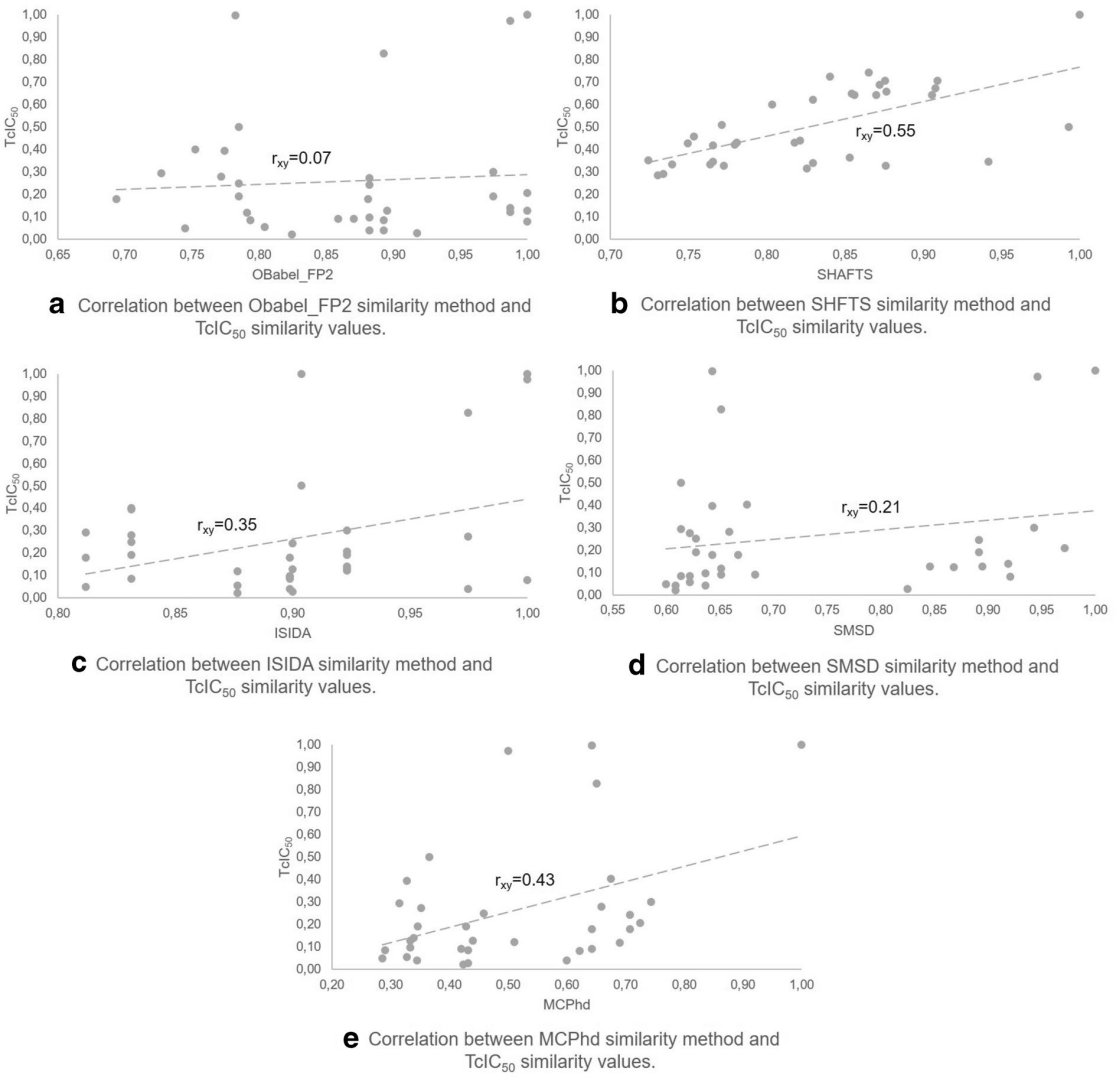
Correlation between similarity methods and $TcIC_{50}$ similarity values between compound 7j and the rest of the dataset

A similar behavior was observed in the results obtained for the less active compound (7j). The slope of MCPhd was closer to $TcIC_{50}$ compared to the other methods (Fig. 12). The $r_{xy}$ = 0.43 of MCPhd vs. $TcIC_{50}$ was higher than the other similarity methods, with the exception of the SHAFTS method where $r_{xy}$ = 0.55 (see Fig. 13).

To generalize these results, the similarity obtained with all methods of the rest of the 17 compounds selected as active by Baptista [44] was correlated against $TcIC_{50}$. The results showed (Table 7) that overall the MCPhd method improved the correlation coefficient in 6% of the cases with respect to SHAFTS and 18% of the cases with respect to the remaining methods.

**Table 7:** Results of correlation between similarity methods and TcIC50 similarity values for the active compounds in the dataset

Molecule	${\hbox {IC}}_{50}$	Correlation				
		${\hbox {r}}_{\mathrm{xy}}$ a	${\hbox {r}}_{\mathrm{xy}}$ b	${\hbox {r}}_{\mathrm{xy}}$ c	${\hbox {r}}_{\mathrm{xy}}$ d	${\hbox {r}}_{\mathrm{xy}}$ e
8c	0.05	0.54	0.66	0.49	0.54	0.84
7c	0.06	0.29	0.62	0.37	0.06	0.80
8f	0.09	0.24	0.60	0.33	0.50	0.48
8l	0.09	0.26	0.62	0.04	0.44	0.55
6c	0.106	− 0.11	0.10	− 0.03	− 0.13	0.64
8b	0.12	0.50	− 0.06	0.49	0.45	0.44
7l	0.17	0.03	0.08	− 0.07	− 0.03	0.36
6i	0.18	− 0.37	0.11	− 0.20	− 0.16	0.25
8i	0.18	0.26	0.64	0.33	0.46	0.56
8d	0.19	0.22	− 0.13	0.33	0.27	− 0.15
8e	0.19	0.23	0.29	0.33	0.38	0.13
8h	0.20	0.24	0.25	0.33	0.35	0.12
8a	0.24	0.50	− 0.11	0.49	0.33	− 0.07
7f	0.25	− 0.05	0.15	0.20	0.02	0.37
7b	0.26	0.24	− 0.09	0.37	− 0.04	0.35
7i	0.26	− 0.03	0.60	0.20	− 0.01	0.40
7h	0.28	− 0.05	0.57	0.20	− 0.13	− 0.02

a-OBabel_FP2 vs ${\hbox {TcIC}}_{50}$, b-SHAFTS vs ${\hbox {TcIC}}_{50}$, c-ISIDA vs ${\hbox {TcIC}}_{50}$,

d-SMSD vs ${\hbox {TcIC}}_{50}$ and e-MCPhd ${\hbox {vsTcIC}}_{50}$

To perform a more exhaustive study comparing the molecular similarity results obtained by all methods, the following steps were performed: (1) the similarity is calculated with all methods for all compounds (one against all); (2) the results up to or equal to the similarity thresholds (0.90, 0.80 and 0.70) are selected for each method; and (3) in each method, the threshold with the highest percentage of success in finding structures with the same activity is selected as the best threshold, and its results are compared.

**Table 8:** Comparison of the observed and predicted by OBabel_FP2 for several similarity thresholds

Threshold	Real-Predicted	Pairs	%	Predicted	Pairs	%
0.90	Active-active	42	31	Correct	77	56
	Inactive-inactive	35	26			
	Active-inactive	32	23	Incorrect	60	44
	Inactive-active	28	20			
	Total	137	100	Total	137	100
0.80	Active-active	101	32	Correct	170	55
	Inactive-inactive	69	22			
	Active-inactive	65	21	Incorrect	141	45
	Inactive-active	76	24			
	Total	311	100	Total	311	100
0.70	Active-active	133	24	Correct	277	49
	Inactive-inactive	144	26			
	Active-inactive	86	15	Incorrect	287	51
	Inactive-active	201	36			
	Total	564	100	Total	564	100

Pairs-Number of predicted pairs

**Table 9:** Comparison of the observed and predicted by SHAFTS for several similarity thresholds

Threshold	Real-predicted	Pairs	%	Predicted	Pairs	%
0.90	Active-active	19	24	Correct	31	42
	Inactive-inactive	13	18			
	Active-inactive	18	24	Incorrect	43	58
	Inactive-active	25	34			
	Total	74	100	Total	74	100
0.80	Active-active	75	29	Correct	137	53
	Inactive-inactive	62	24			
	Active-inactive	43	17	Incorrect	120	47
	Inactive-active	77	30			
	Total	257	100	Total	257	100
0.70	Active-active	107	22	Correct	224	45
	Inactive-inactive	117	24			
	Active-inactive	76	15	Incorrect	271	55
	Inactive-active	195	39			
	Total	495	100	Total	495	100

Pairs-Number of predicted pairs

As a result, a threshold of 0.90 was selected for the OBabel_FP2 method because 56% of the 137 pairs of structures found presented the same activity (active-active and inactive-inactive); for the remaining methods: SHAFTS, ISIDA, SMSD and MCPhd, thresholds of 0.80, 0.90, 0.90 and 0.70 were selected because they presented 53%, 55%, 65% and 67% of pairs of structures with the same activity respectively. Tables 8, 9, 10, 11 and 12 show the results that validate the selection.

**Table 10:** Comparison of the observed and predicted by ISIDA for several similarity thresholds

Threshold	Real-predicted	Pairs	%	Predicted	Pairs	%
0.90	Active-active	94	32	Correct	165	55
	Inactive-inactive	71	24			
	Active-inactive	57	19	Incorrect	133	45
	Inactive-active	76	26			
	Total	298	100	Total	298	100
0.80	Active-active	136	23	Correct	289	49
	Inactive-inactive	153	26			
	Active-inactive	84	14	Incorrect	306	51
	Inactive-active	222	37			
	Total	595	100	Total	595	100
0.70	Active-active	136	23	Correct	289	49
	Inactive-inactive	153	26			
	Active-inactive	84	14	Incorrect	306	51
	Inactive-active	222	37			
	Total	595	100	Total	595	100

Pairs-Number of predicted pairs

**Table 11:** Comparison of the observed and predicted by SMSD for several similarity thresholds

Threshold	Real-predicted	Pairs	%	Predicted	Pairs	%
0.90	Active-active	39	34	Correct	75	65
	Inactive-inactive	36	31			
	Active-inactive	19	17	Incorrect	41	35
	Inactive-active	22	19			
	Total	116	100	Total	116	100
0.80	Active-active	52	28	Correct	106	57
	Inactive-inactive	54	29			
	Active-inactive	48	26	Incorrect	81	43
	Inactive-active	33	18			
	Total	187	100	Total	187	100
0.70	Active-active	63	30	Correct	121	57
	Inactive-inactive	58	27			
	Active-inactive	48	23	Incorrect	91	43
	Inactive-active	43	20			
	Total	212	100	Total	212	100

Pairs-Number of predicted pairs

If we analyze the results obtained with the best similarity thresholds in each method, we can infer that the percentage of structures found with the same activity (active-active and inactive-inactive) obtained with the MCPhd method (67%) was better than the results with the OBabel_FP2, SHAFTS, ISIDA and SMSD methods by 11%, 14%, 12% and 2% respectively. Analyzing only the active-active pairs, the increase was 14%, 16%, 13% and 11% (45% for MCPhd, 31% for Obabel_FP2, 29% for SHAFTS, 32% for ISIDA and 34% for SMSD). These results proved, once again, that the MCPhd method improved the similarity results obtained by the rest of similarity methods studied.

**Fig. 14:**
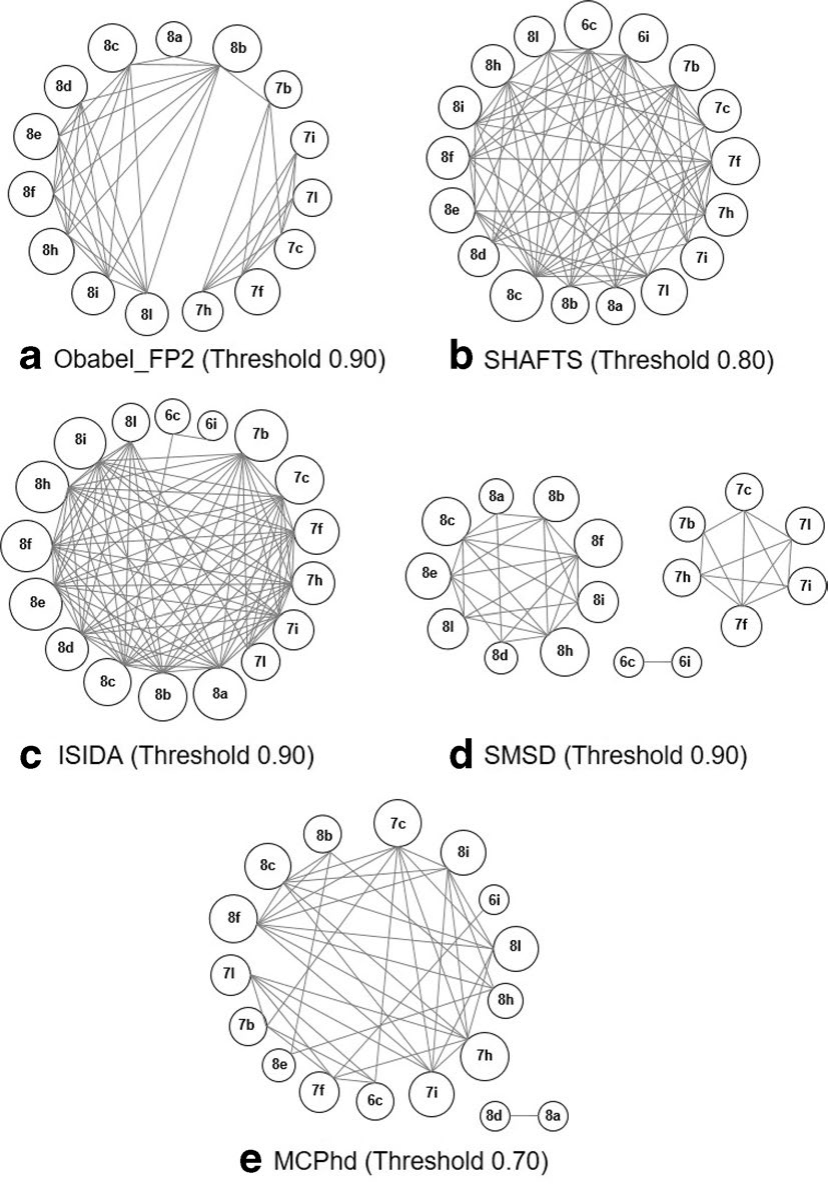
Relationship graph of active compounds with a molecular similarity higher than the selected threshold

As another criterion, the 42 (OBabel_FP2), 75 (SHAFTS), 94 (ISIDA), 39 (SMSD) and 41 (MCPhd) pairs of compounds classified in the active-active category shown in Tables 8, 9, 10, 11 , 12 were compared for all methods, using the best thresholds. To do so, only the 17 active compounds in the dataset were consided and, relationship graphs were drawn for the compounds present in the active-active pairs for each method. Figure 14 shows this representation.

**Table 12:** Comparison of the observed and predicted by MCPhd for several similarity thresholds

Threshold	Real-predicted	Pairs	%	Predicted	Pairs	%
0.90	Active-active	14	33	Correct	26	62
	Inactive-inactive	12	29			
	Active-inactive	8	19	Incorrect	16	38
	Inactive-active	8	19			
	Total	42	100	Total	42	100
0.80	Active-active	16	36	Correct	28	62
	Inactive-inactive	12	27			
	Active-inactive	9	20	Incorrect	17	38
	Inactive-active	8	18			
	Total	45	100	Total	45	100
0.70	Active-active	41	45	Correct	62	67
	Inactive-inactive	23	25			
	Active-inactive	15	16	Incorrect	30	33
	Inactive-active	15	16			
	Total	92	100	Total	92	100

Pairs-Number of predicted pairs

If we observe the differences between the families of compounds 6, 7 and 8 (see Table 1), these differences were fundamentally due to the characteristics of the side chain. Moreover, the hybrid descriptors used in MCPhd have demonstrated [35, 45] their capability to distinguish between the same DC at different positions in a molecule. That allows MCPhd to find relationships/groups of compounds that show a higher functional relationship, and find similarities between compounds of different families. The rest of the compared methods did not show this capacity. As shown in Fig. 14, whereas MCPhd did not relate compounds 8d and 8a with the rest, suggesting that the electrostatic characteristics evidenced by the Electrotopographic State Index for Atoms was the source of the difference these two compounds. SMSD split this sample by grouping the three families separately, because it considers only structural features. SHAFTS considered that all compounds were related, and ISIDA identified similarity between 6c and 6i and the first of this pair with 8c.

This result implies that the MCPhd method allowed establishing similarity relations between compounds even from different families, making logical associations in contrast to the results of the other methods. The reason is that in addition to the structural information content provided by the electrotopographic state index for atoms, it includes the electrostatic information content.

**Fig. 15:**
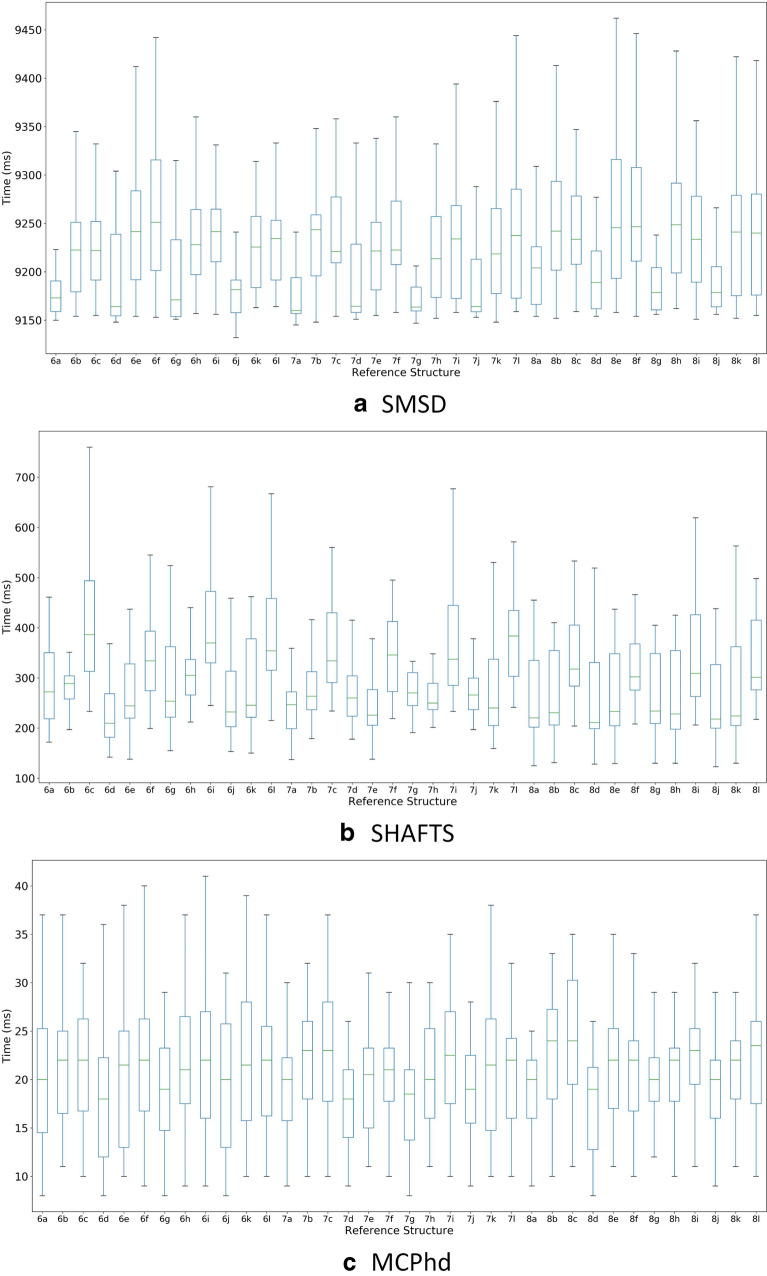
Calculation times

As a last criterion, a runtime comparison was carried out for methods SMSD, SHAFTS and MCPhd. Figure 15 shows the box-plot representation of the computation time when calculating the similarity of each compound (reference structure) with the rest of the dataset. Moreover, the average runtime for SMSD was between 7688.2 and 8770.9 milliseconds, for SHAFTS was between 238.3 and 431.6 milliseconds. In contrast, the average calculation times for the MCPhd were between 18.6 and 35.6 milliseconds.

MCPhd uses a reduced graph, mapping smaller sized molecular graphs. In addition, the similarity calculated by the MCPhd method is based on the criterion of analogy or proximity between the physicochemical properties of the molecular fragments or subgroups that are compared by expressing these properties as a value of $Sstate_{3D}$. On the other hand, SMSD and SHAFTS performs a more expensive mapping process for the compared molecular structures.

## Conclusions

This work proposed a new approach that uses the 3D structure of molecules with physicochemical information to estimate the molecular similarity between chemical compounds. The method has been favorably compared with the standard SMSD, OBabel_FP2, ISIDA and SHAFTS methods and shows better performance in obtaining structures with the same activity using similarity cutoff values during the screening process. Furthermore, the proposal shows the ability to find similar compounds among different families. This strongly suggest the possibility of employing the MCPhd method for isosteric studies.

Finally, the proposal presented in this paper provides a promising method for extending this method to be used in the construction of QSAR models for molecular activity prediction.

## Supplementary information

**Additional file 1.** rar-file containing all necessary data/files to reproduce the results presented in this work.

## Data Availability

All the data on which the conclusions of the work are based have been exhaustively presented in the manuscript. The algorithm implementation for free use, the dataset used in the paper, and other files needed to reproduce the results are included as supplementary material. The source code under GNU General Public License v3.0 can be downloaded from the GitHub repository at the following link: https://github.com/aantelo00/MCPhd.
